# The Vitamin D Metabolite Diagnostic Ratio Associates With Phenotypic Traits of Idiopathic Hypercalciuria

**DOI:** 10.1016/j.ekir.2024.01.004

**Published:** 2024-01-10

**Authors:** Nasser A. Dhayat, Cédric Mattmann, Harald Seeger, Alexander Ritter, Thomas Ernandez, Catherine Stoermann-Chopard, Florian Buchkremer, Stephan Segerer, Beat Roth, Gregoire Wuerzner, Carsten A. Wagner, Olivier Bonny, Albrecht W. Popp, Bruno Vogt, Matteo Bargagli, Daniel G. Fuster

**Affiliations:** 1 B. Braun Medical Care AG, Nephrology and Dialysis Care Center, Hochfelden, Zürich, Switzerland; 2 Swiss National Centre of Competence in Research Kidney.CH, Zürich, Switzerland; 3 Department of Nephrology and Hypertension, Inselspital, Bern University Hospital and University of Bern, Bern, Switzerland; 4 Division of Nephrology, University Hospital Zürich, Zürich, Switzerland; 5 Service of Nephrology, Geneva University Hospitals, Geneva, Switzerland; 6 Division of Nephrology, Kantonsspital Aarau, Aarau, Switzerland; 7 Department of Urology, Lausanne University Hospital, CHUV, University of Lausanne, Switzerland; 8 Service of Nephrology and Hypertension, Lausanne University Hospital and University of Lausanne, Lausanne, Switzerland; 9 Institute of Physiology, University of Zürich, Zürich, Switzerland; 10 Department of Biomedical Sciences, University of Lausanne, Lausanne, Switzerland; 11 Service of Nephrology, Fribourg State Hospital and University of Fribourg, Fribourg, Switzerland; 12 Department of Osteoporosis, Inselspital, Bern University Hospital and University of Bern, Bern, Switzerland; 13 Department of Urology, Inselspital, Bern University Hospital and University of Bern, Bern, Switzerland

**Keywords:** calcium, CYP24A1, kidney stones, nephrolithiasis, vitamin D

## Abstract

**Introduction:**

Underlying mechanisms for hypercalciuria remain unknown in most cases; thus, the designation “idiopathic.” We hypothesized that the vitamin D-inactivating enzyme, CYP24A1 contributes to the pathogenesis of hypercalciuria in kidney stone formers.

**Methods:**

We conducted association analyses between CYP24A1 activity, estimated by the vitamin D metabolite diagnostic ratio (25(OH) vitamin D_3_/total 24,25 (OH)_2_ vitamin D ratio; VMDR), and the phenotype of participants in 2 observational cohorts of kidney stone formers, the Swiss Kidney Stone Cohort (SKSC) and the Bern Kidney Stone Registry (BKSR). Circulating 25(OH)- and 24,25 (OH)_2_ vitamin D were quantified using a validated liquid chromatography tandem mass spectrometry assay.

**Results:**

A total of 974 participants were included in the analysis. We found a positive association of VMDR (and hence negative association of CYP24A1 activity) with total (β 0.009 mmol/l; 95% confidence interval [CI]: 0.002, 0.016; *P* = 0.02) and ionized plasma calcium (β 0.005 mmol/l; 95% CI: 0.002, 0.008; *P* < 0.01), absolute and fractional excretion of urinary calcium (β 0.054 mmol/24h; 95% CI: 0.010, 0.097; *P* = 0.02 and β 0.046%; 95% CI: 0.018, 0.074; *P* < 0.01, respectively). Further, VMDR was associated with an increased likelihood of forming calcium oxalate dihydrate stones (Odds ratio [OR] 1.64; 95% CI: 1.22, 2.35; *P* < 0.01) and reduced bone mineral density (BMD) at the femoral neck (β −0.005 g/cm^2^; 95% CI: −0.010, −0.001; *P* = 0.04). The described associations became stronger when the analysis was confined to idiopathic calcium stone formers.

**Conclusion:**

Our study reveals that CYP24A1 activity, estimated by VMDR, is associated with clinical traits previously linked to idiopathic hypercalciuria.

## Graphical abstract

**Figure:**
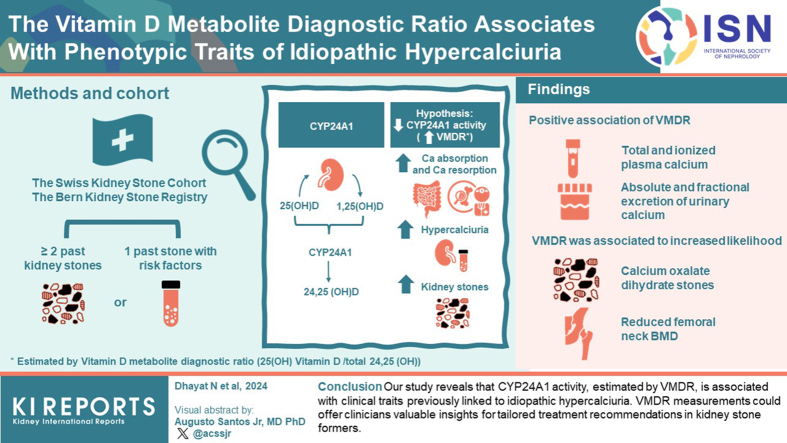

See Commentary on Page 743

Kidney stone formation is complex and depends on dietary, environmental, and genetic factors.^1^ Eighty percent to 90% of stones are composed of calcium oxalate, calcium phosphate, or a mixture of both.^2^^,^^3^ Hypercalciuria, defined as increased urinary calcium excretion, is the most frequent prolithogenic abnormality encountered in patients with kidney stones and is present in up to 80% of affected patients.^3^^,^^4^ Metabolic work-up of hypercalciuric stone formers rarely reveals an underlying systemic cause, and thus in most cases, hypercalciuria is defined as “idiopathic.”^5^ The phenotype of idiopathic hypercalciuria can be replicated by exogenous administration of 1,25(OH)_2_ vitamin D_3_ to healthy individuals.^6^ In contrast, the P450 inhibitor ketoconazole, which attenuates 1,25(OH)_2_ vitamin D_3_ synthesis, normalized the clinical features of patients with idiopathic hypercalciuria.^7^ Circulating 1,25(OH)_2_ vitamin D_3_ is determined by the rate of synthesis from its substrate, 25(OH) vitamin D_3_, through the activity of 25(OH)D-1-hydroxylase (CYP27B1), and its degradation by 1,25(OH)_2_D-24-hydroxylase (CYP24A1).^8^ CYP24A1 is a P450 enzyme expressed in target tissues and is strongly up-regulated by its substrate 1,25(OH)_2_ vitamin D_3_. In addition to 1,25(OH)_2_ vitamin D_3_, CYP24A1 also inactivates its precursor 25(OH) vitamin D. Homozygous pathogenic variants in *CYP24A1* cause idiopathic infantile hypercalcemia, a condition first described in children undergoing vitamin D supplementation characterized by failure to thrive, dehydration, hypercalcemia, and nephrocalcinosis.^9^ Subsequently, biallelic pathogenic variants in *CYP24A1* were also discovered in adults with elevated vitamin D, suppressed parathyroid hormone (PTH), hypercalcemia, hypercalciuria, osteopenia, and recurrent calcium nephrolithiasis.^10^^,^^11^ Individuals with heterozygous pathogenic *CYP24A1* variants exhibit an intermediary phenotype characterized by recurrent calcium nephrolithiasis, normocalcemic hypercalciuria, high-normal 1,25(OH)_2_ vitamin D_3_, and low-normal PTH, a constellation typically encountered in idiopathic hypercalciuria, suggesting a gene dosage effect for pathogenic *CYP24A1* variants.^10^^,^^12^^,^^13^

The ratio between 25(OH)- and 24,25(OH)_2_ vitamin D (VMDR) is considered as a proxy of CYP24A1-mediated vitamin D clearance.^13^, ^14^, ^15^ In a cohort of 153 first-time calcium stone formers, increased serum calcium and 1,25(OH)_2_ vitamin D_3_, a lower serum 24,25(OH)_2_ vitamin D / 25(OH) vitamin D ratio but no difference in urine calcium was observed compared to non-stone formers.^16^

The association of CYP24A1 activity, estimated by the VMDR, with stone composition and comprehensive clinical traits linked to kidney stone disease, has not been studied yet. Therefore, the diagnostic utility of the VMDR in kidney stone formers remains unclear. Given the high phenotypic similarity between carriers of pathogenic *CYP24A1* variants and kidney stone formers with idiopathic hypercalciuria, we hypothesized that reduced CYP24A1 activity may be an important cause of idiopathic hypercalciuria. To this end, we assessed the association of VMDR with the clinical phenotype of participants in 2 large prospective observational Swiss cohorts of kidney stone formers.

## Methods

### Study Population

The SKSC is an investigator-initiated prospective, multicenter, observational study of patients with kidney stones recruited at the nephrology outpatient clinics of 6 tertiary care nephrology centers in Switzerland (Aarau, Basel, Bern, Geneva, Lausanne, and Zürich).^17^ The BKSR includes kidney stone formers recruited at the nephrology outpatient clinic of the Department of Nephrology and Hypertension at the Bern University Hospital, Bern, Switzerland.^18^ SKSC and BKSR adhered to the Declaration of Helsinki and were approved by the responsible cantonal ethical committees (approval #BE 173/13 and #BE 95/06, respectively). Inclusion criteria for both cohorts are written informed consent, age ≥18 years, ≥2 past kidney stone events or 1 past stone event combined with additional risk factors for stone recurrence, such as first stone episode <25 years, positive family history, stones other than calcium oxalate, bilateral or multiple stones or nephrocalcinosis detected by imaging, single kidney or chronic kidney disease (estimated glomerular filtration rate <60 ml/min), metabolic syndrome, gout, or osteoporosis. A stone event was defined as a symptomatic stone event with visible passage of a stone (with or without accompanying typical symptoms), or urological intervention of a symptomatic or asymptomatic stone. At the baseline visit, demographic and anthropometric data, comorbidities and stone composition analysis results were collected, and a comprehensive blood and 24-hour urine metabolic workup performed. In addition, biobank plasma samples were collected at the baseline visit, immediately frozen and stored at −80 °C. Stone formers with end stage kidney disease were excluded from this study. A total of 974 participants (560 SKSC participants and 414 BKSR participants) met the eligibility criteria and had biobank plasma available for the analysis of vitamin D metabolites.

### Measurements and Definitions

Plasma 25(OH) vitamin D_2_ and D_3,_ and total plasma 24,25(OH)_2_ vitamin D were measured by an established, highly sensitive and specific liquid chromatography tandem mass spectrometry method at the Mayo Clinic Central Laboratories, Rochester, MN.^19^^,^^20^ 25(OH) vitamin D_2_ was not detected in any of the participants, thus the VMDR was calculated as 25(OH) vitamin D_3_/total 24,25(OH)_2_ vitamin D. All other blood and urinary parameters of BKSR and SKSC participants were measured centrally by standard clinical laboratory methods at the Central Laboratory of the Bern University Hospital, Bern, Switzerland. Assay characteristics for the measurements of PTH, C-terminal fibroblast growth factor 23 (cFGF23) and 1,25(OH)_2_ vitamin D_3_ were previously described.^21^ Estimated glomerular filtration rate was determined with the Chronic Kidney Disease-Epidemiology Collaboration 2009 equation.^22^ The mean value of two 24-hour urine collections on consecutive days was used to calculate mean 24-hour urinary calcium excretion. Fractional excretion of calcium was determined from blood and urine samples collected on the same day. Osteodensitometry at the lumbar spine and the femoral neck was performed in all BKSR participants at the time point of metabolic work-up at the Department of Osteoporosis of the Bern University Hospital, Bern, Switzerland, by dual-energy X-ray absorptiometry (DEXA; Hologic QDR 4500A, Hologic, Bedford, MA).^23^ Kidney stone composition was determined by Fourier-transform infrared spectroscopy. In participants with several stone composition analyses available, the most recent analysis prior to metabolic work-up was included in the analysis. Urine relative supersaturations of calcium oxalate and brushite (calcium phosphate) were calculated using the EQUIL2 program.^24^ Data related to loop and thiazide diuretics and medications that could potentially influence plasma 25(OH) vitamin D_3_ concentration were collected. These included vitamin D supplementation (cholecalciferol, ergocalciferol, calcifediol, and alfacalcidol), carbamazepine, oxcarbazepine, clonazepam, St. John’s wort, ritonavir, efavirenz, tenofovir, emtricitabine, glucocorticoids, rifampin, ketoconazole, and calcium antagonists (felodipine, amlodipine, nifedipine, lercanidipine). Noncalcium stones were defined as stone composition containing ≥50% uric acid, struvite, or cystine.

### Statistical Analyses

Continuous variables were reported as medians ± interquartile ranges or means ± SD. Categorical variables were reported as counts and percentages, as appropriate. All values were first analyzed by descriptive statistics. All statistical tests were 2-sided, and a *P*-value <0.05 was considered statistically significant. Unadjusted and adjusted linear and logistic regression analyses of CYP24A1 activity, measured by the VMDR, were conducted as predictor variables with appropriately transformed outcome variables. Age, sex, body mass index, estimated glomerular filtration rate, and plasma 25(OH) vitamin D_3_ were considered potential confounders and included in the multivariable model. These variables were selected for their known interaction with the outcome variables and total plasma 24,25(OH)_2_ vitamin D concentration by affecting 1,25(OH)_2_ vitamin D_3_ synthesis. To further assess the association between clinical traits of idiopathic hypercalciuria and the VMDR in patients with or without low 25(OH) vitamin D_3_, we stratified the study population in 2 subgroups (<20 or ≥20 ng/ml). In addition, we performed sensitivity analyses for medications (drugs modulating plasma 25(OH) vitamin D_3_ concentration, loop and thiazide diuretics), urinary sodium excretion, which can directly affect urinary calcium excretion and total calcium balance,^25^ and for month of VMDR measurement, to account for fluctuations related to seasonality. A fully-adjusted subgroup analysis was conducted specifically on idiopathic calcium stone formers. This excluded patients without available stone composition analysis, those with noncalcium stones, and patients with secondary forms of calcium stones.^26^ None of the participants included in this study had an established diagnosis of infantile hypercalcemia. All continuous predictor and outcome variables were scaled to Z-scores. The standardized regression coefficients (β) were subsequently multiplied by the SD of the corresponding outcome variable. Therefore, the resulting β coefficients are interpreted as the expected change, in absolute values, of the outcome variable per each SD increase in the predictor variable (VMDR), which corresponds to 0.47. All regression models were tested for residuals’ normality and homoscedasticity using visual inspection and for highly influential observations by plotting the Cook’s distance for each data point. Residual plots were generated and carefully examined for normality through histograms along with Q-Q plots, whereas for homoscedasticity, a scatter plot of the predicted values against the residuals was inspected. All plots displayed expected patterns, suggesting that the assumptions of normality and equal variances were reasonably met for our models. Further, all analyses were checked for incomplete or over-collected 24-hour urine samples using reference values for urinary 24-hour creatinine excretion based on the adult Swiss population.^27^ None of the results reported were biased by collection adequacy. Statistical analyses were conducted using the R software, version 4.2.1.^28^

## Results

### Characteristics of the Study Population

A total of 974 kidney stone formers met the predefined eligibility criteria and were included in the analysis. Baseline characteristics of the study population are shown in Table 1. Overall, 69.5% of participants (*n* = 677) were men. Mean age ± SD and age at the first kidney stone event ± SD were 47.5 ± 14.4 and 36.9 ± 14.2 years, respectively. The majority of participants (*n* = 762, 81%) were recurrent stone formers. A stone composition analysis was available in 77% (*n* = 751) of participants. The most abundant stone type in our cohort (defined as ≥50% of total stone content) was calcium oxalate monohydrate (54%) as reported previously,^29^ followed by calcium oxalate dihydrate (18%), total calcium phosphate (12%), and uric acid (8%).

**Table 1:** Baseline characteristics of study population

Characteristics	Study participants (*N* = 974)
Males	677 (69.5%)
Age, y	47.5 (14.4)
Age at first self-reported stone event, y	36.9 (14.2)
Age at first stone composition analysis, y	45.8 (14.6)
Body mass index, kg/m^2^	27.1 (4.9)
eGFR creatinine Equation CKD-EPI 2009, ml/min per 1.73 m^2^ BSA	95.0 (19.7)
Hypertension medication usage	268 (27.6%)
Diabetes	114 (11.7%)
Stone recurrence (≥2 stone events)	793 (81.3%)
Medications affecting plasma 25(OH) vitamin D3	71 (7.3%)
Loop diuretics	14 (1.4%)
Thiazide diuretics	127 (13.0%)
DEXA parameters	
Femoral neck BMD, g/cm^2^	0.83 (0.75, 0.93)
Femoral neck T-score, SD	−0.60 (−1.30, 0.00)
Lumbar spine BMD, g/cm^2^	1.01 (0.93, 1.11)
Lumbar spine T-score, SD	−0.60 (−1.40, 0.30)
Blood parameters	
Total plasma calcium, mmol/l	2.34 (2.28, 2.39)
Corrected plasma calcium, mmol/l	2.36 (2.30, 2.42)
Ionized calcium, mmol/l	1.20 (1.17, 1.22)
Phosphate, mmol/l	0.99 (0.87, 1.11)
25(OH) vitamin D_3_, ng/ml	21.00 (14.00, 29.00)
1,25(OH)_2_ vitamin D_3_, pmol/l	99.00 (74.79, 127.00)
Total 24,25(OH)_2_ vitamin D ng/ml	1.46 (0.73, 2.30)
VMDR	14.78 (11.51, 19.67)
Intact PTH, ng/l	39.00 (30.60, 50.00)
cFGF23, RU/ml	69.50 (54.35, 96.00)
Urinary parameters	
Urinary calcium, mmol/24h	5.14 (3.33, 7.48)
Fractional excretion of calcium, %	2.72 (1.79, 3.57)
RSS for calcium oxalate	4.08 (2.05, 7.62)
RSS for brushite	0.74 (0.27, 2.06)
Relative kidney stone composition ≥50%	
Available stone composition analysis	751 (77.1%)
Total calcium oxalate	595 (79.2%)
Calcium oxalate monohydrate	399 (54.0%)
Calcium oxalate dihydrate	132 (17.9%)
Total calcium phosphate	91 (12.1%)
Apatite	63 (8.4%)
Brushite	15 (2.0%)
Uric acid	60 (8.0%)
Cystine	9 (1.2%)

BKSR, Bern Kidney Stone Registry; BMD, bone mineral density; BSA, body surface area; cFGF23, C-terminal fibroblast growth factor 23; CKD-EPI, Chronic Kidney Disease-Epidemiology Collaboration; DEXA, dual-energy X-ray absorptiometry; eGFR, estimated glomerular filtration rate; PTH, parathyroid hormone; SKSC, Swiss Kidney Stone Cohort; VMDR, Vitamin D metabolite diagnostic ratio.

Characteristics are indicated for all participants enrolled from the BKSR and the SKSC**.** Categorical variables are described by number of participants *N* (%), continuous variables are described by their mean (SD) or median (25^th^–75^th^ percentile).

**Figure 1:**
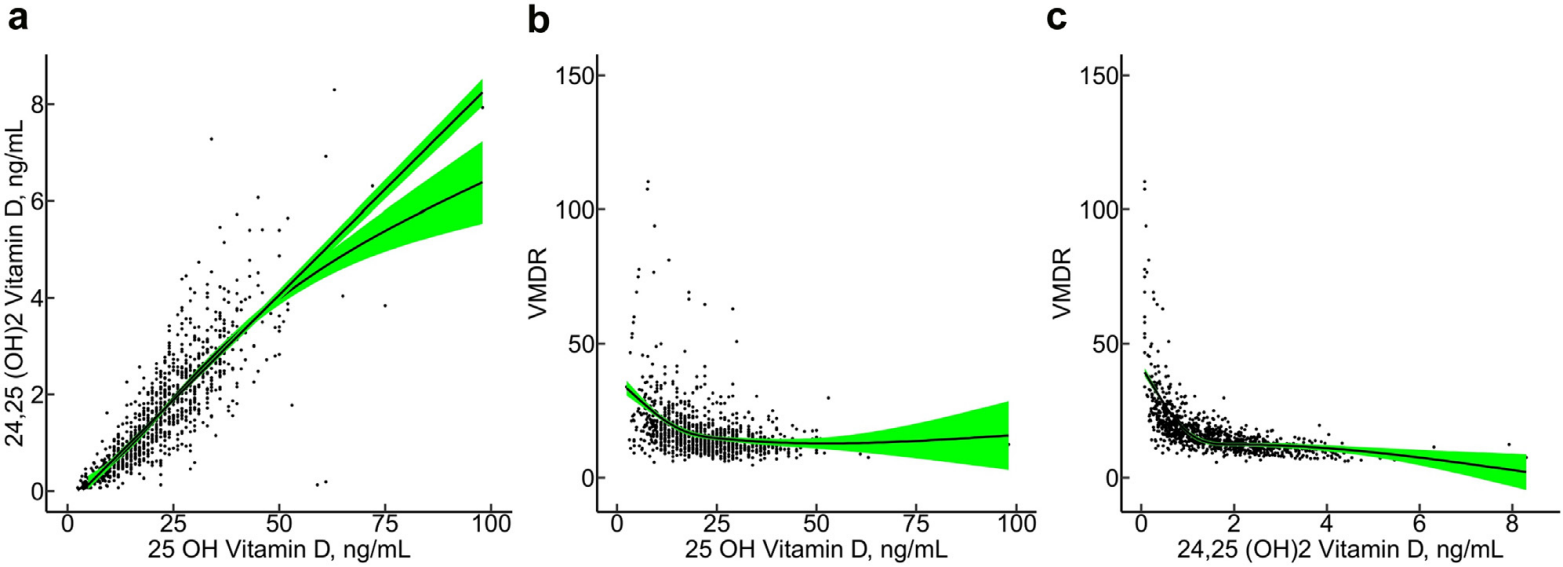
Associations between 25(OH) vitamin D_3_, total 24,25(OH)_2_ vitamin D and VMDR. Panel (a) shows a scatterplot of the association between 25(OH) vitamin D_**3**_ and total 24,25(OH)_2_ vitamin D levels, including linear and natural cubic spline regression lines with green shadowed areas representing the 95% confidence bands. The association appears to be approximately linear at 25(OH) vitamin D_**3**_ concentration between 0 to 50 ng/ml, and total 24,25(OH)_2_ vitamin D between 0 to 4 ng/ml. Panel (b) shows the association between 25(OH) vitamin D_**3**_ and VMDR restricted to values ≤150 and a natural cubic spline regression line. Panel (c) shows a scatterplot of the association between total 24,25(OH)_2_ vitamin D and VMDR restricted to values ≤150 and a natural cubic spline regression line. VMDR, vitamin D metabolite diagnostic ratio

Median values (interquartile ranges) for plasma 25(OH) vitamin D_3_, total 24,25(OH)_2_ vitamin D, and the VMDR were 21.0 (14.0, 29.0) ng/ml, 1.46 (0.73, 2.30) ng/ml, and 14.8 (11.5, 19.7), respectively (Table 1). There was a strong correlation between 25(OH)- and 24,25(OH)_2_ vitamin D (Pearson’s correlation coefficient = 0.83, *P* < 0.001) (Figure 1a). The VMDR was not normally distributed (Supplementary Figure S1).

A VMDR <25, considered normal (https://www.mayocliniclabs.com/test-catalog/Overview/63416#Clinical-and-Interpretive), was present in 87.1% (*n* = 848) of participants.^19^^,^^20^ A VMDR between 25 and 80, previously reported in monoallelic carriers of pathogenic *CYP24A1* variants, was found in 12.1% (*n* = 118) of participants.^19^ A VMDR >80, previously reported in biallelic carriers of pathogenic *CYP24A1* variants, was found in 0.8% (*n* = 8) participants.^19^ Baseline characteristics stratified by these VMDR cut-offs are shown in Table 2. A replete 25(OH) vitamin D_3_ status (≥20 ng/ml) was found in 56.2% (*n* = 547) of participants. Within this subgroup, 94.9% (*n* = 519) had a VMDR <25, 4.6% (*n* = 25) between 25 and 80, and 0.5% (*n* = 3) >80.

**Table 2:** Blood and urine mineral metabolism parameters and DEXA parameters stratified by subgroups of VMDR

Characteristics	Vitamin D metabolite diagnostic ratio		
	VMDR <25 (*N* = 848)	VMDR 25–80 (*N* = 118)	VMDR >80 (*N* = 8)
Males	604 (71.2%)	68 (57.6%)	5 (62.5%)
Age, years	47.4 (14.2)	47.8 (15.5)	51.1 (15.2)
Age at first self-reported stone event	37.1 (14.1)	36.2 (15.0)	29.3 (9.0)
Age at first stone composition analysis	45.6 (14.3)	46.6 (16.7)	48.3 (17.7)
Body mass index, kg/m^2^	27.0 (4.8)	28.1 (5.4)	24.9 (2.7)
eGFR creatinine Equation CKD-EPI 2009, ml/min per 1.73 m^2^ BSA	95.4 (18.7)	93.6 (25.0)	80.2 (25.4)
Hypertension medication usage	232 (27.4%)	34 (28.8%)	2 (25.0%)
Diabetes	94 (11.1%)	20 (16.9%)	0 (0.0%)
Stone recurrence (≥2 stone events)	689 (81.3%)	96 (81.4%)	8 (100.0%)
Medications affecting plasma 25(OH) vitamin D3	55 (6.5%)	15 (12.7%)	1 (12.5%)
Loop diuretics	11 (1.3%)	3 (2.5%)	0 (0.0%)
Thiazide diuretics	115 (13.6%)	11 (9.3%)	1 (12.5%)
DEXA parameters			
Femoral neck BMD, g/cm^2^	0.84 (0.76, 0.93)	0.79 (0.72, 0.88)	0.79 (0.65, 0.84)
Femoral neck T-score, SD	−0.50 (−1.20, 0.10)	−0.80 (−1.48, −0.20)	−1.05 (−2.00, −0.70)
Lumbar spine BMD, g/cm^2^	1.02 (0.94, 1.11)	0.98 (0.85, 1.08)	1.01 (0.97, 1.06)
Lumbar spine T-score, SD	−0.60 (−1.28, 0.30)	−0.70 (−2.00, 0.18)	−0.70 (-0.98, −0.28)
Blood parameters			
Total plasma calcium, mmol/l	2.34 (2.28, 2.39)	2.32 (2.26, 2.39)	2.43 (2.34, 2.53)
Corrected plasma calcium, mmol/l	2.36 (2.30, 2.42)	2.36 (2.29, 2.43)	2.46 (2.34, 2.54)
Ionized calcium, mmol/l	1.20 (1.17, 1.22)	1.20 (1.18, 1.23)	1.24 (1.21, 1.26)
Phosphate, mmol/l	1.00 (0.88, 1.11)	0.98 (0.87, 1.10)	0.96 (0.91, 1.01)
25(OH) vitamin D_3_, ng/ml	22.00 (16.00, 30.00)	12.00 (8.30, 18.00)	12.50 (9.00, 31.30)
1,25(OH)_2_ vitamin D_3_, pmol/l	100.00 (76.00, 127.00)	90.00 (67.00, 121.00)	81.50 (72.50, 105.00)
Total 24,25(OH)_2_ vitamin D ng/ml	1.65 (0.96, 2.44)	0.37 (0.24, 0.57)	0.12 (0.07, 0.14)
Intact PTH, ng/l	38.60 (30.40, 49.00)	44.00 (32.00, 59.50)	42.00 (21.40, 45.00)
cFGF23, RU/ml	69.30 (54.20, 94.80)	71.10 (55.20, 111.50)	94.80 (65.70, 236.20)
Urine parameters			
Urinary calcium, mmol/24h	5.20 (3.43, 7.48)	4.51 (2.66, 7.15)	9.52 (6.07, 10.30)
Fractional excretion of calcium, %	2.72 (1.84, 3.56)	2.66 (1.55, 3.55)	3.59 (2.95, 5.24)
RSS for calcium oxalate	4.00 (2.00, 7.50)	5.00 (2.70, 8.60)	5.70 (2.60, 7.90)
RSS for brushite	0.70 (0.30, 2.00)	1.00 (0.30, 3.10)	1.30 (0.40, 4.70)
Relative kidney stone composition ≥ 50%			
Total calcium oxalate	525 (79.4%)	65 (77.4%)	5 (83.3%)
Calcium oxalate monohydrate	354 (54.4%)	44 (53.7%)	1 (16.7%)
Calcium oxalate dihydrate	112 (17.2%)	16 (19.5%)	4 (66.7%)
Total calcium phosphate	76 (11.5%)	14 (16.7%)	1 (16.7%)
Apatite	53 (8.0%)	9 (10.7%)	1 (16.7%)
Brushite	12 (1.8%)	3 (3.6%)	0 (0.0%)
Uric acid	57 (8.6%)	3 (3.6%)	0 (0.0%)
Cystine	8 (1.2%)	1 (1.2%)	0 (0.0%)

BMD, bone mineral density; BSA, body surface area; cFGF23, C-terminal fibroblast growth factor 23; CKD-EPI, Chronic Kidney Disease-Epidemiology Collaboration; DEXA, dual-energy X-ray absorptiometry; eGFR, estimated glomerular filtration rate; PTH, parathyroid hormone; VMDR, Vitamin D metabolite diagnostic ratio.

Categorical variables are described by number of participants *N* (%), continuous variables are described by their mean (SD) or median (25th–75th percentile).

Stone formers with low 25(OH) vitamin D_3_ (<20 ng/ml) had significantly higher VMDR compared to the normal range (Figure 1b), in line with previous studies.^19^^,^^20^ As expected, a negative relationship was observed between 24,25(OH)_2_ vitamin D and VMDR (Figure 1c).

The VMDR exhibited seasonal variability, with a median peak value in February, suggesting the lowest CYP24A1 activity, and with a nadir reached in September, indicating the highest enzyme activity (Supplementary Figure S2, Supplementary Table S1).

### Association Analyses

The unadjusted analysis revealed a significant direct association of VMDR with total and ionized calcium, cFGF23, and fractional excretion of calcium; and an inverse correlation with BMD at the femoral neck (Table 3). After adjusting for multiple confounders, including age, sex, body mass index, estimated glomerular filtration rate, and plasma 25(OH) vitamin D_3_ (Table 3), the results remained broadly consistent, confirming the association between VMDR and total (β 0.009 mmol/l; 95% CI: 0.002, 0.016; *P* = 0.02) and ionized calcium (β 0.005 mmol/l; 95% CI: 0.002, 0.008; *P* < 0.01) , cFGF23 (β 0.045 RU/ml; 95% CI: 0.004, 0.086; *P* = 0.03), fractional excretion of calcium (β 0.046%; 95% CI: 0.018, 0.074; *P* < 0.01) and BMD at the femoral neck (β −0.005 g/cm^2^; 95% CI: −0.010, −0.001; *P* = 0.04). In addition, absolute urinary calcium excretion became directly correlated with VMDR (β 0.054 mmol/24h; 95% CI: 0.010, 0.097; *P* = 0.02). No association was found between VMDR and plasma 1,25(OH)_2_ vitamin D_3_ or PTH. Only after accounting for cFGF23 as an additional confounder, a trend toward a direct association between 1,25(OH)_2_ vitamin D_3_ and VMDR was observed (β 0.131 pmol/l; 95% CI: 0.000, 0.267; *P* = 0.05).

**Table 3:** Univariable and multivariable linear regression analysis

Outcome variable	Univariable Model				Multivariable Model			
	*N*	β	95% CI	*P*-value	*N*	β	95% CI	*P*-value
Blood parameters								
Total plasma calcium, mmol/l	960	0.009	0.002, 0.016	0.031	959	0.009	0.002, 0.016	0.016
Ionized calcium, mmol/l	601	0.005	0.002, 0.009	0.002	599	0.005	0.002, 0.008	0.004
Phosphate, mmol/l	962	−0.003	−0.014, 0.008	0.570	961	−0.004	−0.015, 0.007	0.460
b1,25(OH)_2_ vitamin D_3_, pmol/l	956	−0.052	−0.174, 0.07	0.400	953	0.029	−0.082, 0.139	0.610
aIntact PTH, ng/l	942	0.019	−0.007, 0.045	0.150	939	0.008	−0.018, 0.033	0.560
bcFGF23, RU,ml	356	0.065	0.023, 0.108	0.002	352	0.045	0.004, 0.086	0.030
Urinary parameters								
aUrinary calcium, mmol/24h	947	0.022	−0.023, 0.067	0.330	944	0.054	0.010, 0.097	0.015
bFractional excretion of calcium, %	931	0.041	0.013, 0.069	0.004	931	0.046	0.018, 0.074	0.001
aRSS for calcium oxalate	801	0.042	−0.046, 0.129	0.350	807	0.068	−0.017, 0.154	0.118
aRSS for brushite	812	0.077	−0.062, 0.217	0.277	796	0.128	−0.005, 0.261	0.059
DEXA parameters								
bLumbar BMD, g/cm^2^	358	−0.002	−0.007, 0.003	0.470	352	−0.001	−0.006, 0.004	0.650
bFemoral BMD, g/cm^2^	359	−0.008	−0.013, −0.002	0.008	353	−0.005	−0.010, −0.001	0.036

BMD, bone mineral density; BMI, body mass index; eGFR, estimated glomerular filtration rate; PTH, parathyroid hormone; RSS, relative supersaturation

Number of observations (*N* obs), beta coefficients (β), 95% confidence intervals (95% CI) and *P*-values are indicated for each comparison.

Association between vitamin D metabolite diagnostic ratio with clinical traits as outcome variables, adjusted for age, sex, BMI, eGFR and 25(OH) vitamin D_3_.

a natural logarithm transformed

b square root transformed

Further, our analysis revealed an inverse relationship between VMDR and the OR to develop calcium oxalate monohydrate-containing stones (Table 4), whereas the likelihood of forming calcium oxalate stones in general was not associated with VMDR. Specifically, a lower CYP24A1 activity, as reflected by a higher VMDR, was associated with a decreased odds of forming stones composed of ≥50% calcium oxalate monohydrate (adjusted OR 0.56; 95% CI: 0.38, 0.79; *P* < 0.01). In contrast, VMDR was associated with an increased odds of stones with a calcium oxalate dihydrate component ≥50% (adjusted OR 1.64; 95% CI: 1.22, 2.35; *P* < 0.01). No correlations between VMDR and relative supersaturations of calcium oxalate or relative supersaturations of brushite were observed.

**Table 4:** Univariable and multivariable logistic regression analysis

Outcome variables	Univariable Model				Multivariable Model			
Relative kidney stone composition ≥ 50%	*N* obs	OR	95% CI	*P*-value	*N* obs	OR	95% CI	*P*-value
Total calcium oxalate	751	0.912	0.737, 1.142	0.370	745	0.961	0.775, 1.237	0.720
Calcium oxalate monohydrate	739	0.656	0.469, 0.873	0.009	733	0.558	0.377, 0.785	0.002
Calcium oxalate dihydrate	739	1.406	1.103, 1.887	0.015	733	1.636	1.217, 2.353	0.004
Total calcium phosphate	751	1.170	0.924, 1.476	0.150	745	1.152	0.906, 1.493	0.220
Apatite	751	1.158	0.879, 1.463	0.200	745	1.104	0.845, 1.454	0.420
Brushite	751	1.128	0.546, 1.505	0.550	745	1.235	0.479, 1.836	0.500

BMI, body mass index; eGFR, estimated glomerular filtration rate.

Number of observations (*N* obs), Odds ratios (OR), 95% confidence intervals (95% CI) and *P*-values are indicated for each comparison.

Univariable and multivariable association between Vitamin D metabolite diagnostic ratio with relative kidney stone composition analysis ≥ 50%. Multivariable model adjusted for age, sex, BMI, eGFR and 25(OH) vitamin D_3_.

### Sensitivity Analyses

Sodium intake is an important determinant of urinary calcium excretion,^25^ and medications that modulate plasma 25(OH) vitamin D_3_ or diuretics may potentially affect the VMDR or urine calcium, respectively. In addition, we found a mild seasonal variability of VMDR. To address these issues, we performed sensitivity analyses that incorporated the month of VMDR measurement, 24-hour urinary sodium excretion, an established proxy of sodium intake, along with the aforementioned medications. All the associations found remained robust in these sensitivity analyses (Supplementary Tables S2–S7).

Further sensitivity analyses were conducted to examine the persistence of associations in subgroups of participants with varying plasma 25(OH) vitamin D_3_ levels. In stone formers with normal plasma 25(OH) vitamin D_3_ (≥20 ng/ml), the aforementioned adjusted associations remained significant (Supplementary Table S8). In contrast, in patients with low 25(OH) vitamin D_3_ (<20 ng/ml), adjusted associations remained valid only for ionized calcium (β 0.01 mmol/l; 95% CI: 0.00, 0.02; *P* = 0.04) and its fractional excretion (β 0.08 %; 95% CI: 0.02, 0.14; *P* < 0.01).

### Confirmatory Analysis for Idiopathic Calcium Stone Formers

To confirm these results in the selected population of interest, we conducted a fully-adjusted analysis on idiopathic calcium stone formers (Tables 5 and 6). In this analysis, we excluded noncalcium stone formers, patients without available stone analysis, and those with secondary causes of calcium stones. Consistent with our main analysis, this subgroup exhibited significant direct associations of VMDR with total plasma calcium (β 0.014 mmol/l; 95% CI: 0.003, 0.025; *P* = 0.011), ionized calcium (β 0.009 mmol/l; 95% CI: 0.001, 0.016; *P* = 0.025), absolute (β 0.077 mmol/24h; 95% CI: 0.018, 0.136; *P* = 0.011), and fractional urinary excretion of calcium (β 0.051 %; 95% CI: 0.01, 0.093; *P* = 0.016) . The inverse association with femoral BMD was also confirmed (β −0.018 g/cm2; 95% CI: −0.030, −0.007; *P* = 0.002).

**Table 5:** Confirmatory analysis for idiopathic calcium stone formers – linear regression analysis

Outcome variables	*N* obs	Vitamin D Metabolite Diagnostic Ratio		
		β	95% CI	*P*-value
Blood parameters				
Total plasma calcium, mmol/L	606	0.014	0.003, 0.025	0.011
Ionized calcium, mmol/L	379	0.009	0.001, 0.016	0.025
Phosphate, mmol/L	607	−0.007	−0.024, 0.009	0.398
b1,25(OH)_2_ Vitamin D_3_, pmol/L	605	0.060	−0.111, 0.23	0.491
aIntact PTH, ng/L	593	0.002	−0.039, 0.042	0.938
Urinary parameters				
aUrinary calcium, mmol/24h	607	0.077	0.018, 0.136	0.011
bFractional excretion of calcium, %	601	0.051	0.01, 0.093	0.016
aRSS for calcium oxalate	504	0.051	−0.045, 0.148	0.298
aRSS for brushite	501	0.071	−0.076, 0.217	0.345
DEXA parameters				
bLumbar BMD, g/cm^2^	232	−0.007	−0.018, 0.004	0.189
bFemoral BMD, g/cm^2^	233	−0.018	−0.030, −0.007	0.002

BMD, bone mineral density; BMI, body mass index; dRTA, distal renal tubular acidosis; eGFR, estimated glomerular filtration rate; PTH, parathyroid hormone

Number of observations (*N* obs), beta coefficients (β), 95% confidence intervals (95% CI) and *P*-values are indicated for each comparison.

Multivariable association between Vitamin D metabolite diagnostic ratio with clinical traits as outcome variables, adjusted for age, sex, BMI, eGFR, 25(OH) vitamin D_3_, urinary sodium excretion, month of VMDR measurement, loop and thiazide diuretics, and medications that interfere with plasma 25(OH) vitamin D3 concentration. Non-calcium stone formers, patients without available stone analysis and with secondary causes for kidney stones were excluded (primary hyperparathyroidism, sarcoidosis, complete dRTA, primary or enteric hyperoxaluria

a natural logarithm transformed

b square root transformed

**Table 6:** Confirmatory analysis for idiopathic calcium stone formers – logistic regression analysis

Outcome variables	*N* obs	Vitamin D Metabolite Diagnostic Ratio		
		OR	95% CI	*P-*value
Total calcium oxalate	595	0.844	0.668, 1.067	0.157
Calcium oxalate monohydrate	588	0.404	0.258, 0.632	<0.001
Calcium oxalate dihydrate	588	1.62	1.147, 2.289	0.006

BMI, body mass index; dRTA, distal renal tubular acidosis; eGFR, estimated glomerular filtration rate

Multivariable logistic regression between Vitamin D metabolite diagnostic ratio with relative kidney stone composition analysis ≥ 50%. Multivariable model adjusted for age, sex, BMI, eGFR, 25(OH) vitamin D_3_, urinary sodium excretion, loop and thiazide diuretics, and medications that interfere with plasma 25(OH) Vitamin D3 concentration. Non-calcium stone formers, patients without available stone analysis and with secondary causes for kidney stones were excluded (primary hyperparathyroidism, sarcoidosis, complete dRTA, primary or enteric hyperoxaluria). Number of observations (*N* obs), Odds ratios (OR), 95% confidence intervals (95% CI) and *P*-values are indicated for each comparison.

In terms of stone composition, the VMDR was inversely associated with the odds of calcium oxalate monohydrate stones formation (adjusted OR 0.40; 95% CI: 0.26, 0.63; *P* < 0.001), and positively with calcium oxalate dihydrate stones (adjusted OR 1.62; 95% CI: 1.15, 2.29; *P* = 0.006), in line with our results obtained in the full cohort.

## Discussion

High urinary calcium excretion (“hypercalciuria”) is a risk factor for both kidney stone formation and low bone mass. However, the underlying mechanisms remain elusive in most patients; thus, the condition is labelled “idiopathic hypercalciuria.”^3^^,^^4^

Our study conducted in 2 large and deeply phenotyped Swiss cohorts of kidney stone formers now reveals that the VMDR (and hence the activity of CYP24A1 inversely) is directly associated with urinary calcium, the key prolithogenic abnormality in kidney stone formers. In support of this finding, CYP24A1 activity was associated with kidney stone composition; we observed an direct association of VMDR (and hence inverse association with CYP24A1 activity) with calcium oxalate dihydrate stones, the classical stone type of patients with a high urine calcium-to-oxalate ratio.^29^ In contrast, there was an inverse association of VMDR (and thus direct association with CYP24A1 activity) with calcium oxalate monohydrate stones, typically encountered in patients with a high urinary oxalate-to-calcium ratio. Consonant with the function of CYP24A1 as vitamin D-inactivating enzyme, we observed a direct association of VMDR with both total plasma calcium and ionized calcium.

Reduced BMD and an increased fracture risk has been repeatedly observed in kidney stone formers, especially in patients with idiopathic hypercalciuria.^30^^,^^31^ Intriguingly, we also observed that reduced CYP24A1 activity is associated with a lower BMD at the femoral neck. In contrast, VMDR was not associated with BMD at the lumbar spine, indicating that CYP24A1 activity rather affects cortical than trabecular bone. Given the cross-sectional nature of our analysis, we can only speculate on the underlying mechanisms. Despite a vitamin D-mediated increase in intestinal calcium absorption in patients with low CYP24A1 activity, urinary calcium losses may prevail, and thus patients are overall in a negative calcium balance, as previously reported in patients with idiopathic hypercalciuria.^32^ Indeed, urine calcium excretion was found to be inversely correlated with BMD changes at the femoral neck but not at the lumbar spine in patients with idiopathic hypercalciuria.^32^ Alternatively, given the known function of 24,25 (OH)_2_ vitamin D in bone remodeling, cell migration, and proliferation,^33^, ^34^, ^35^ low levels of 24,25 (OH)_2_ vitamin D may directly be responsible for reduced BMD observed at the femoral neck.^36^

Results of our analyses in kidney stone formers reveal that CYP24A1 activity, estimated by the VMDR, correlates with clinical traits characteristic of idiopathic hypercalciuria after adjustment for multiple confounders, including plasma 25(OH) vitamin D_3_ and drugs that modulate its concentration, loop and thiazide diuretics, and urinary sodium excretion. Notably, the aforementioned associations became even stronger when only idiopathic calcium stone formers were analyzed. However, these observations appear only valid in patients which are 25(OH) vitamin D_3_ replete, whereas with 25(OH) vitamin D_3_ deficiency (<20 ng/ml), the VMDR will be falsely elevated.

Surprisingly, we observed a significant correlation between CYP24A1 activity and cFGF23, but no association was evident with plasma PTH and 1,25 (OH)_2_ vitamin D_3_. Of note, only 13 participants exhibited increased serum ionized calcium, and the majority of our cohort did not demonstrate overt hypercalcemia, which may have further weakened the association between PTH and reduced CYP24A1 activity. Furthermore, there was only a very weak correlation between 1,25 (OH)_2_ vitamin D_3_ and urinary calcium excretion in our study population (Spearman’s rho 0.11; *P* < 0.01). FGF23 plays a pivotal role in the regulation of vitamin D metabolism by decreasing circulating 1,25 (OH)_2_ vitamin D_3_ through CYP27B1 inhibition and CYP24A1 induction in the kidney.^37^ Individuals with infantile hypercalcemia type 1 and very low CYP24A1 activity have high FGF23 levels.^38^^,^^39^ In our analysis, a trend toward an association between lower CYP24A1 activity and higher 1,25 (OH)_2_ vitamin D_3_ was observed after adjusting for FGF23. Thus, we speculate that reduced CYP24A1 activity might lead to higher FGF23, which in turn reduces 1,25 (OH)_2_ vitamin D_3_ synthesis through inhibition of CYP27B1. However, 1,25 (OH)_2_ vitamin D_3_ is also regulated by PTH as part of a complex interplay between calcium, 25(OH) vitamin D_3_, and PTH. In an observational study, such intricate relationships may be challenging to assess, and reverse causality or alternative explanations, such as tissue-specific nonsystemic effects of CYP24A1 or a 1,25(OH)2 vitamin D3–independent regulation of renal calcium handling by CYP24A1 cannot be excluded. Consonant with this, kidney stone formers were recently shown to exhibit higher CYP24A1 expression in circulating monocytes compared to nonstone formers when individual 1,25(OH)2 vitamin D_3_ concentrations were taken into account (i.e., higher circulating 1,25 (OH)_2_ vitamin D_3_ /monocyte CYP24A1 expression ratios).^40^

According to previous reports, a VMDR >80 suggests biallelic pathogenic *CYP24A1* variants, whereas values between 25 and 80 have been reported in heterozygous carriers or in patients with low plasma 25(OH) vitamin D_3_.^10^^,^^19^ The prevalence of pathogenic variants in *CYP24A1* in the general population has been estimated between 420 and 1960 per 100,000 individuals (0.4 to 2%).^11^ The observation that 28 of 547 (5 %) of vitamin D-replete kidney stone formers in our cohort had a VMDR >25 indicates that other factors may play an important role in the regulation of CYP24A1 activity. Indeed, CYP24A1 expression is regulated by a myriad of endocrine factors, including PTH, FGF23, 1,25 (OH)_2_ vitamin D_3_, estrogens, and retinoid receptor ligands as well as inflammatory cytokines such as IL-6 and TNF-α.^41^, ^42^, ^43^, ^44^, ^45^, ^46^ Furthermore, epigenetic modifications at the *CYP24A1* promoter and tissue-specific intronic enhancer modulate transcription of the *CYP24A1* gene.^47^^,^^48^ Therefore, the VMDR employed in this study integrates both genetic and nongenetic factors influencing CYP24A1 activity. As such, our measurements of the VMDR in both idiopathic calcium-kidney stone formers and unselected kidney stone formers therefore offer a comprehensive view of biochemical and clinical traits associated with CYP24A1 activity. Additional strengths of our study include the large sample size, the multicenter study design, and a very detailed phenotype including stone analysis and BMD measurements. Our study also has several limitations, including the observational design, the exploratory nature of the analyses, an almost exclusive Caucasian study population, the lack of genetic data, and absence of low risk first-time kidney stone formers in the study population.

Despite these limitations, our analyses significantly extend current knowledge on the role of the vitamin D-inactivating enzyme CYP24A1 in nephrolithiasis. Our results demonstrate that CYP24A1 activity, estimated by VMDR, is directly linked to urine calcium excretion, kidney stone composition, and BMD at the femoral neck. Future studies are now needed to confirm the validity of VMDR as a diagnostic tool in the metabolic work-up of kidney stone formers and to determine if VMDR status prospectively predicts risk of recurrence and is associated with the response to dietary and pharmacologic preventive strategies. If validated, VMDR measurements could offer clinicians valuable insights for tailored treatment recommendations in kidney stone formers.

## Disclosure

CAW reports honoraria from Kyowa Kirin and Advicenne. OB was a consultant for Otsuka, CSL Vifor, Alexion, Bayer, Alnylam, Kyowa Kirin, and Boehringer Ingelheim; and received unrestricted research grants from Otsuka and Alnylam. AR received support for attending meetings and/or travel expenses from Salmon Pharma, Astellas Pharma, and Boehringer Ingelheim and honoraria from CSL Vifor. DGF served as a consultant for Otsuka, Alnylam, Boehringer Ingelheim, and Kyowa Kirin and received unrestricted research grants from Otsuka, Boehringer Ingelheim, and CSL Vifor. All the other authors declared no competing interests.

## Supplementary Material

**Supplementary Methods.**

**Formulas.**

**Figure S1.** Distribution of VMDR.

**Figure S2.** Seasonal variation of 25(OH) vitamin D3, 24,25(OH)2 vitamin D3, and their ratio.

**Table S****1****.** Seasonal variability of VMDR.

**Table** **S****2****.** Sensitivity linear regression analysis for urinary sodium excretion.

**Table S****3****.** Sensitivity logistic regression analysis for urinary sodium excretion.

**Table S****4****.** Sensitivity linear regression analysis for medications.

**Table S****5****.** Sensitivity logistic regression analysis for medications.

**Table S****6****.** Sensitivity linear regression analysis for seasonality of VMDR measurement.

VMDR, vitamin D metabolite diagnostic ratio

**Table S****7****.** Sensitivity logistic regression analysis for seasonality of VMDR measurement.

VMDR, vitamin D metabolite diagnostic ratio

**Table S****8****.** Multivariable linear regression analysis stratified by plasma 25(OH) Vitamin D3.

**Table S9.** Missing variables report.

**STROBE Statement.**
