# Supplementary material for: The Vitamin D Metabolite Diagnostic Ratio Associates With Phenotypic Traits of Idiopathic Hypercalciuria
Source: Kidney Int Rep. 2024 Jan 10;9(4):1072–82. doi: 10.1016/j.ekir.2024.01.004 (PMC11101794; doi:10.1016/j.ekir.2024.01.004)
Supplement: Supplementary File (PDF) [file mmc1.pdf]

SUPPLEMENTARY MATERIAL

Supplementary Methods

Formulas

The calculation of corrected plasma calcium ($Ca_{corr)}$ was performed by the formula: Ca_{corr} (mmol/L) = measured plasma calcium (mmol/L) + 0.025 × (40 – plasma albumin (g/L)), in case of plasma albumin ≤40 g/L.

Fractional excretion of calcium (FeCa) and phosphate (FePi) were calculated using the following formulas:

FeCa = (24h urinary Ca * plasma creatinine / plasma ionized Ca * 24h urinary creatinine) * 100

Plasma ionized calcium rather than total calcium was computed for the calculation of FeCa to ensure a more accurate reflection of bioavailable calcium.

Supplementary Table S1 | Seasonal variability of VMDR. Seasonal variability per season and month of VMDR, 25(OH) Vitamin D_3 and total 24,25$(OH)_2$ Vitamin D**.** Variables are described by their median (25th-75th percentile). VMDR, Vitamin D metabolite diagnostic ratio.

	**VMDR**	**25(OH) Vitamin D_3 (ng/mL)**	**24,25$(OH)_2$ Vitamin D (ng/mL)**
**Season**			
Spring	15.97 (13.15, 21.02)	17.50 (11.00, 25.00)	1.04 (0.58, 1.84)
Summer	14.14 (11.41, 18.67)	26.00 (21.00, 33.00)	1.85 (1.18, 2.60)
Autumn	13.24 (10.73, 17.36)	24.00 (18.00, 30.00)	1.88 (1.13, 2.67)
Winter	16.15 (12.33, 20.87)	15.00 (10.00, 23.50)	0.98 (0.50, 1.76)
**Month**			
January	15.87 (12.32, 21.28)	14.00 (9.75, 21.50)	0.98 (0.51, 1.71)
February	17.91 (13.59, 23.46)	14.00 (9.10, 21.00)	0.68 (0.38, 1.44)
March	16.77 (13.27, 21.48)	17.00 (10.75, 25.00)	1.02 (0.53, 1.83)
April	15.70 (12.63, 20.86)	17.00 (11.00, 23.25)	0.90 (0.55, 1.68)
May	14.86 (13.21, 20.89)	20.00 (14.00, 27.00)	1.20 (0.69, 2.07)
June	15.58 (12.10, 19.88)	24.00 (18.75, 30.25)	1.59 (1.02, 2.48)
July	13.58 (11.37, 18.44)	25.00 (21.00, 31.00)	1.91 (1.11, 2.53)
August	13.27 (10.95, 16.22)	29.50 (24.00, 37.00)	2.22 (1.64, 3.24)
September	12.32 (10.33, 16.08)	27.00 (23.00, 33.75)	2.04 (1.57, 2.97)
October	13.20 (10.66, 17.96)	24.00 (21.00, 30.00)	1.99 (1.14, 2.69)
November	14.73 (11.55, 19.63)	19.50 (15.00, 29.00)	1.33 (0.78, 2.17)
December	13.79 (10.95, 17.89)	17.00 (12.50, 26.00)	1.33 (0.82, 2.15)

Supplementary Table S2 | Sensitivity linear regression analysis for urinary sodium excretion. Association between Vitamin D metabolite diagnostic ratio with clinical traits as outcome variables, adjusted for age, sex, BMI, eGFR, 25(OH) vitamin D_3 and 24h urinary sodium excretion. BMI, body mass index; eGFR, estimated glomerular filtration rate; PTH, parathyroid hormone; BMD, bone mineral density; RSS, relative supersaturation; *, natural logarithm transformed; **, square root transformed. Number of observations (N obs), beta coefficients (β), 95% confidence intervals (95% CI) and *p*-values are indicated for each comparison.

		Vitamin D Metabolite Diagnostic Ratio		
**Outcome variables**	**N obs**	**β**	**95% CI**	***p*-value**
**Blood parameters**				
Total plasma calcium, mmol/L	939	0.009	0.002, 0.016	0.022
Ionized calcium, mmol/L	592	0.005	0.002, 0.009	0.005
Phosphate, mmol/L	939	-0.003	-0.014, 0.008	0.510
**1,25$(OH)_2$ Vitamin D_3, pmol/L	935	-0.052	-0.174, 0.070	0.560
*Intact PTH, ng/L	920	0.019	-0.007, 0.045	0.590
**cFGF23, RU,mL	346	0.065	0.023, 0.108	0.032
**Urinary parameters**				
*Urinary calcium, mmol/24h	943	0.022	0.018, 0.067	0.038
**Fractional excretion of calcium, %	930	0.041	0.013, 0.069	0.002
*RSS for calcium oxalate	791	0.042	-0.046, 0.129	0.111
*RSS for brushite	780	0.077	-0.062, 0.217	0.060
**Bone parameters**				
**Lumbar BMD, g/cm^2	348	-0.002	-0.007, 0.003	0.660
**Femoral BMD, g/cm^2	349	-0.008	-0.013, -0.002	0.037

Supplementary Table S3 | Sensitivity logistic regression analysis for urinary sodium excretion. Multivariable association between Vitamin D metabolite diagnostic ratio with relative kidney stone composition analysis ≥ 50%. Multivariable model adjusted for age, sex, BMI, eGFR , 25(OH) vitamin D_3 and urinary sodium excretion. BMI, body mass index; eGFR, estimated glomerular filtration rate. Number of observations (N obs), Odds ratios (OR), 95% confidence intervals (95% CI) and *p*-values are indicated for each comparison.

		**Vitamin D Metabolite Diagnostic Ratio**		
**Outcome variables**	**N obs**	**OR**	**95% CI**	***p*-value**
Total calcium oxalate	728	0.961	0.773, 1.237	0.720
Calcium oxalate monohydrate	716	0.565	0.382, 0.794	0.002
Calcium oxalate dihydrate	716	1.609	1.204, 2.311	0.005
Total calcium phosphate	728	1.140	0.894, 1.475	0.260
Apatite	728	1.105	0.844, 1.444	0.410
Brushite	728	1.204	0.411, 1.859	0.600

Supplementary Table S4 | Sensitivity linear regression analysis for medications. Association between Vitamin D metabolite diagnostic ratio with clinical traits as outcome variables, adjusted for age, sex, BMI, eGFR, 25(OH) vitamin D3, loop and thiazide diuretics, and medications that interfere with plasma 25(OH) Vitamin D3 concentration. BMI, body mass index; eGFR, estimated glomerular filtration rate; PTH, parathyroid hormone; BMD, bone mineral density; *, natural logarithm transformed; **, square root transformed. Number of observations (N obs), beta coefficients (β), 95% confidence intervals (95% CI) and p-values are indicated for each comparison.

	Vitamin D Metabolite Diagnostic Ratio			
**Outcome variables**	**N obs**	**β**	**95% CI**	***p*-value**
**Blood parameters**				
Total plasma calcium, mmol/L	959	0.009	0.002, 0.016	0.008
Ionized calcium, mmol/L	599	0.005	0.001, 0.008	0.005
Phosphate, mmol/L	961	-0.004	-0.015, 0.006	0.434
**1,25(OH)$_2$ Vitamin D$_3$, pmol/L	953	0.028	-0.083, 0.138	0.624
*Intact PTH, ng/L	939	0.008	-0.017, 0.033	0.539
**cFGF23, RU,mL	352	0.047	0.006, 0.088	0.025
**Urinary parameters**				
*Urinary calcium, mmol/24h	944	0.055	0.012, 0.098	0.013
**Fractional excretion of calcium, %	931	0.046	0.019, 0.074	0.001
*RSS for calcium oxalate	807	0.069	-0.016, 0.155	0.111
*RSS for brushite	796	0.128	-0.005, 0.261	0.059
**DEXA parameters**				
**Lumbar BMD, g/cm^2	352	-0.001	-0.006, 0.004	0.638
**Femoral BMD, g/cm^2	353	-0.006	-0.011, -0.001	0.030

Supplementary Table S5 | Sensitivity logistic regression analysis for medications. Multivariable association between Vitamin D metabolite diagnostic ratio with relative kidney stone composition analysis ≥ 50%. Multivariable model adjusted for age, sex, BMI, eGFR , 25(OH) vitamin D_3, loop and thiazide diuretics, and medications that interfere with plasma 25(OH) Vitamin D3 concentration. BMI, body mass index; eGFR, estimated glomerular filtration rate. Number of observations (N obs), Odds ratios (OR), 95% confidence intervals (95% CI) and *p*-values are indicated for each comparison.

		Vitamin D Metabolite Diagnostic Ratio		
**Outcome variables**	**N obs**	**OR**	**95% CI**	***p*-value**
Total calcium oxalate	745	0.956	0.773, 1.181	0.674
Calcium oxalate monohydrate	733	0.543	0.374, 0.786	0.001
Calcium oxalate dihydrate	733	1.664	1.186, 2.335	0.003
Total calcium phosphate	745	1.172	0.936, 1.468	0.167
Apatite	745	1.131	0.890, 1.437	0.313
Brushite	609	1.211	0.595, 2.465	0.597

Supplementary Table S6 | Sensitivity linear regression analysis for seasonality of VMDR measurement. Association between Vitamin D metabolite diagnostic ratio with clinical traits as outcome variables, adjusted for age, sex, BMI, eGFR, 25(OH) vitamin D3 and month of VMDR measurement. BMI, body mass index; eGFR, estimated glomerular filtration rate; PTH, parathyroid hormone; BMD, bone mineral density; *, natural logarithm transformed; **, square root transformed. Number of observations (N obs), beta coefficients (β), 95% confidence intervals (95% CI) and p-values are indicated for each comparison.

		Vitamin D Metabolite Diagnostic Ratio		
**Outcome variables**	**N obs**	**β**	**95% CI**	***p*-value**
**Blood parameters**				
Total plasma calcium, mmol/L	959	0.009	0.003, 0.016	0.007
Ionized calcium, mmol/L	599	0.005	0.002, 0.008	0.004
Phosphate, mmol/L	961	-0.003	-0.014, 0.007	0.534
**1,25(OH)$_2$ Vitamin D$_3$, pmol/L	953	0.024	-0.086, 0.135	0.667
*Intact PTH, ng/L	939	0.006	-0.019, 0.032	0.636
**cFGF23, RU,mL	352	0.051	0.009, 0.092	0.016
**Urinary parameters**				
*Urinary calcium, mmol/24h	944	0.048	0.004, 0.092	0.031
**Fractional excretion of calcium, %	931	0.043	0.015, 0.071	0.003
*RSS for calcium oxalate	807	0.073	-0.014, 0.159	0.099
*RSS for brushite	796	0.132	-0.004, 0.267	0.056
**DEXA parameters**				
**Lumbar BMD, g/cm^2	352	-0.002	-0.007, 0.003	0.409
**Femoral BMD, g/cm^2	353	-0.006	-0.011, -0.001	0.024

Supplementary Table S7 | Sensitivity logistic regression analysis for seasonality of VMDR measurement. Multivariable association between Vitamin D metabolite diagnostic ratio with relative kidney stone composition analysis ≥ 50%. Multivariable model adjusted for age, sex, BMI, eGFR , 25(OH) vitamin D_3 and month of VMDR measurement. BMI, body mass index; eGFR, estimated glomerular filtration rate. Number of observations (N obs), Odds ratios (OR), 95% confidence intervals (95% CI) and *p*-values are indicated for each comparison.

		**Vitamin D Metabolite Diagnostic Ratio**		
**Outcome variables**	**N obs**	**OR**	**95% CI**	***p*-value**
Total calcium oxalate	745	0.976	0.786, 1.212	0.826
Calcium oxalate monohydrate	733	0.557	0.383, 0.811	0.002
Calcium oxalate dihydrate	733	1.639	1.175, 2.287	0.004
Total calcium phosphate	745	1.166	0.924, 1.470	0.195
Apatite	745	1.118	0.871, 1.435	0.383
Brushite	573	1.134	0.572, 2.249	0.719

Supplementary Table S8 | Multivariable linear regression analysis stratified by plasma 25(OH) Vitamin D_3. Association between the Vitamin D metabolite diagnostic ratio with clinical traits as outcome variables, stratified by plasma 25(OH) vitamin D_3 concentration and adjusted for age, sex, BMI, eGFR and 24h urinary sodium excretion. BMI, body mass index; eGFR, estimated glomerular filtration rate; PTH, parathyroid hormone; *, natural logarithm transformed; **, square root transformed. Number of observations (N), beta coefficients (β), 95% confidence intervals (95% CI) and *p*-values are indicated for each comparison.

	**25(OH) Vitamin D < 20 ng/mL (N = 427)**			**25(OH) Vitamin D ≥ 20 ng/mL (N = 547)**		
**Outcome variables**	**β**	**95% CI**	***p*-value**	**β**	**95% CI**	***p*-value**
**Blood parameters**						
Total plasma calcium, mmol/L	0.01	-0.00, 0.03	0.124	0.01	0.001, 0.02	0.030
Ionized calcium, mmol/L	0.01	0.00, 0.02	0.039	0.00	0.00, 0.01	0.033
Phosphate, mmol/L	0.01	-0.02, 0.03	0.603	0.00	-0.02, 0.01	0.515
**1,25(OH)$_2$ Vitamin D_3, pmol/L	0.10	-0.14, 0.3	0.407	0.07	-0.06, 0.19	0.318
*Intact PTH, ng/L	0.01	-0.05, 0.10	0.675	0.00	-0.03, 0.03	0.986
**cFGF23, RU,mL	0.00	-0.11, 0.11	0.847	0.05	0.01, 0.09	0.015
**Urinary parameters**						
*Urinary calcium, mmol/24h	0.06	-0.02, 0.14	0.164	0.05	0.00, 0.10	0.049
**Fractional excretion of calcium, %	0.08	0.02, 0.14	0.009	0.04	0.01, 0.07	0.016
*RSS for calcium oxalate	0.12	-0.02, 0.26	0.102	0.03	-0.08, 0.15	0.574
*RSS for brushite	0.24	-0.22, 0.66	0.243	-0.01	-0.2, 0.2	0.867
**Bone parameters**						
**Lumbar BMD, g/cm^2	0.00	-0.01, 0.01	0.860	0.00	-0.01, 0.01	0.783
**Femoral BMD, g/cm^2	-0.01	-0.02, 0.01	0.374	-0.01	-0.01, -0.00	0.042

Supplementary Table 9 | Missing variables report. Missing values for each variable analyzed in the study are indicated for all participants enrolled from the BKSR and the SKSC**.** Variables are described as absolute N (%). BSA, body surface area; eGFR, estimated glomerular filtration rate; DEXA, dual-energy X-ray absorptiometry; PTH, parathyroid hormone; BMD, bone mineral density; RSS, relative supersaturation.

**Characteristics**	**Overall missing variables**
Males	0 (0.0%)
Age, years	0 (0.0%)
Body mass index, kg/m^2	0 (0.0%)
eGFR creatinine Equation CKD-EPI 2009, mL/min per 1.73 m^2 BSA	9 (0.9%)
Medications affecting plasma 25(OH) Vitamin D3	0 (0.0%)
Loop diuretics	0 (0.0%)
Thiazide diuretics	0 (0.0%)
**DEXA parameters**	
Femoral neck BMD, g/cm^2	616 (63.2%)
Lumbar spine BMD, g/cm^2	615 (63.1%)
**Blood parameters**	
Total plasma calcium, mmol/L	14 (1.4%)
Ionized calcium, mmol/L	373 (38.3%)
Phosphate, mmol/L	12 (1.2%)
1,25(OH)$_2$ Vitamin D$_3$, pmol/L	18 (1.9%)
Intact PTH, ng/L	32 (3.3%)
cFGF23, RU/mL	618 (63.5%)
**Urinary parameters**	
Urinary calcium, mmol/24h	27 (2.8%)
Fractional excretion of calcium, %	43 (4.4%)
RSS for calcium oxalate	162 (16.6%)
RSS for brushite	173 (17.8%)
**Kidney stone composition**	
No available stone composition analysis	223 (22.9%)

Supplementary Figures

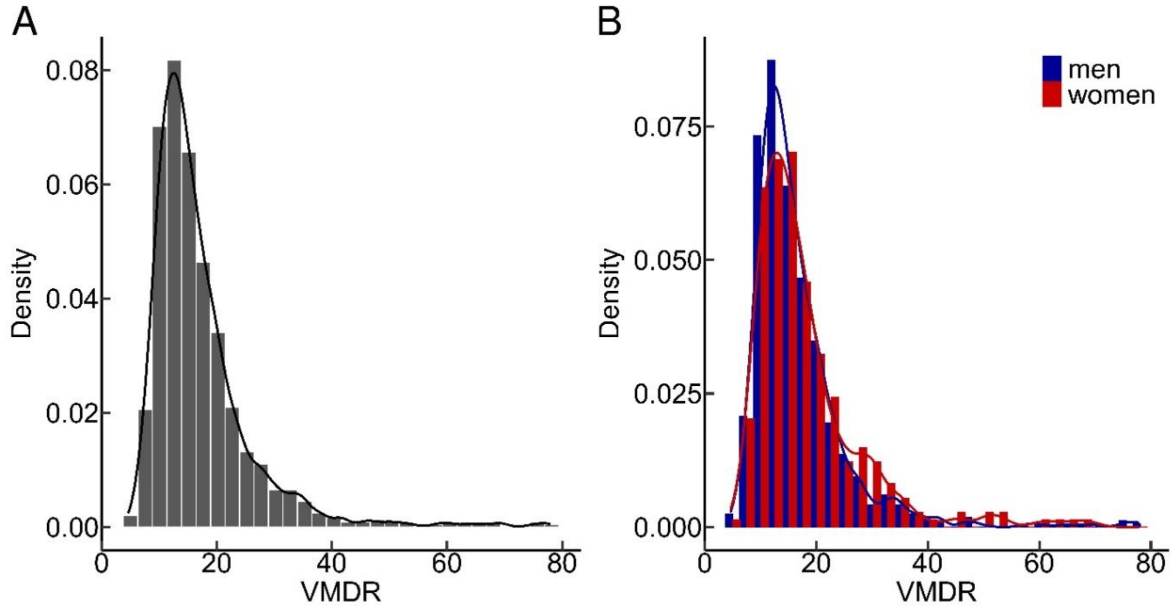

Supplementary Figure 1. Distribution of VMDR. Panel (A) and (B) show density plots representing the probability density function for the kernel density estimation with overlaid histograms corresponding to frequencies of VMDR (Vitamin D metabolite diagnostic ratio; 25(OH) Vitamin D_3 / total $24,25(OH)_2$ Vitamin D ratio) for the whole cohort (panel A) and separated by sex (panel B). Displayed values are restricted to VMDR < 80 to enhance visibility.

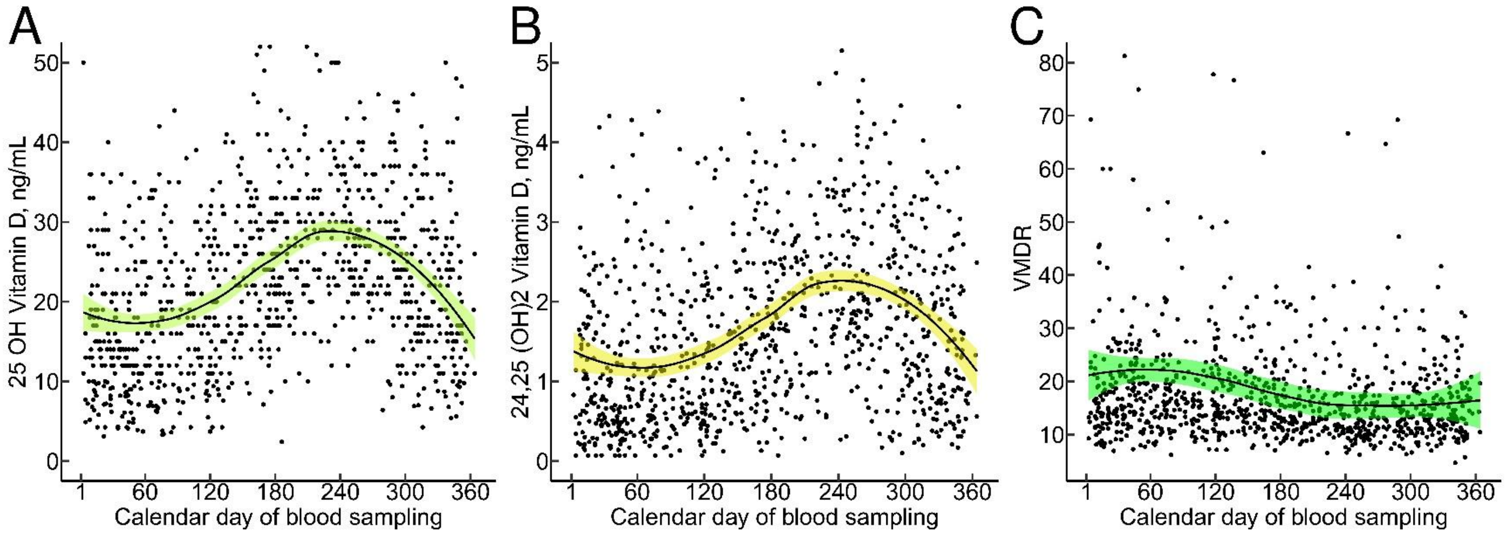

Supplementary Figure 2. Seasonal variation of 25(OH) Vitamin D_3, 24,25$(OH)_2$ Vitamin D_3 and their ratio. Scatterplots of the association between calendar days of sampling on the x-axes, and 25(OH) Vitamin D_3 (panel A), total 24,25$(OH)_2$ Vitamin D (panel B), and VMDR (Vitamin D metabolite diagnostic ratio; 25(OH) Vitamin D_3 / 24,25$(OH)_2$ Vitamin D ratio) (panel C) on the y-axes. Locally Weighted Scatterplot Smoothing (LOWESS) lines with shadowed areas representing the 95% confidence bands are shown for each panel. Displayed values were restricted to enhance visibility.

Modified STROBE Statement—checklist of items that should be included in reports of observational studies (Cohort/Cross-sectional and case-control studies)

	Item No	Recommendation
**Title and abstract**	1	(*a*) Indicate the study's design with a commonly used term in the title or the abstract.<br><br>**Page 3 of manuscript (Abstract)**
		(*b*) Provide in the abstract an informative and balanced summary of what was done and what was found<br><br>**Page 3 of manuscript (Abstract)**
**Introduction**		
Background/rationale	2	Explain the scientific background and rationale for the investigation being reported<br><br>**Pages 5 and 6 of manuscript (Introduction)**
Objectives	3	State specific objectives, including any prespecified hypotheses<br><br>**Page 5 and 6 of manuscript (Introduction)**
**Methods**		
Study design	4	Present key elements of study design early in the paper<br><br>**Page 3 of manuscript (Abstract)**<br><br>**Pages 7, 8 and 9 of manuscript (Methods)**
Setting	5	Describe the setting, locations, and relevant dates, including periods of recruitment, exposure, follow-up, and data collection<br><br>**Pages 7 and 8 of manuscript (Methods)**
Participants	6	*Cohort study*—Give the eligibility criteria, and the sources and methods of selection of participants. Describe methods of follow-up<br><br>**N/A**<br><br>*Case-control study*—Give the eligibility criteria, and the sources and methods of case ascertainment and control selection. Give the rationale for the choice of cases and controls<br><br>**N/A**

		Cross-sectional study—Give the eligibility criteria, and the sources and methods of selection of participants<br><br>**Page 7 of manuscript (Methods)**
Variables	7	Clearly define all outcomes, exposures, predictors, potential confounders, and effect modifiers. Give diagnostic criteria, if applicable<br><br>**Pages 7, 8 and 9 of manuscript (Methods)**
Data sources/ measurement	8*	For each variable of interest, give sources of data and details of methods of assessment (measurement).<br><br>**Pages 7, 8 and 9 of manuscript (Methods)**
Bias	9	Describe any efforts to address potential sources of bias<br><br>**Pages 7, 8 and 9 of manuscript (Methods)**
Study size	10	Explain how the study size was arrived at (if applicable)<br><br>**N/A**
Quantitative variables	11	Explain how quantitative variables were handled in the analyses. If applicable, describe which groupings were chosen and why<br><br>**Pages 7-10 of manuscript (Methods)**
Statistical methods	12	(*a*) Describe all statistical methods, including those used to control for confounding<br><br>**Pages 7-10 of manuscript (Methods)**
		(*b*) Describe any methods used to examine subgroups and interactions<br><br>**Pages 7-10 of manuscript (Methods)**
		(*c*) Explain how missing data were addressed<br><br>**Pages 25-30 of manuscript (Tables 1,2,3 and 4) and pages 8 -10 of manuscript (Methods). Number of observations indicated.**
		Cohort study—If applicable, explain how loss to follow-up was addressed<br><br>*Case-control study*—If applicable, explain how matching of cases and controls was addressed<br><br>*Cross-sectional study*—If applicable, describe analytical methods taking account of sampling strategy

		(*e*) Describe any sensitivity analyses<br><br>**Supplementary Table S1, S2, S3 and S4. Sensitivity analysis for urinary sodium excretion and medications that can influence plasma 25(OH) Vitamin D_3.**
**Results**		
Participants	13*	(a) Report numbers of individuals at each stage of study—eg numbers potentially eligible, examined for eligibility, confirmed eligible, included in the study, completing follow-up, and analyzed<br><br>**Pages 10-14 of manuscript (Results). Pages 26-30 of manuscript (Tables 1,2,3 and 4).**
		(c) **Use of a flow diagram**<br><br>**Not done, but described in detail in Methods section.**
Descriptive data	14*	(a) Give characteristics of study participants (eg demographic, clinical, social) and information on exposures and potential confounders<br><br>**Table 1**
		(b) Indicate number of participants with missing data for each variable of interest<br><br>**Tables 1-2-3-4.**
		(c) *Cohort study*—Summarise follow-up time (eg, average and total amount)
Outcome data	15*	*Cohort study*—Report numbers of outcome events or summary measures over time<br><br>**N/A**
		Case-control study—Report numbers in each exposure category, or summary measures of exposure<br><br>**N/A**
		Cross-sectional study—Report numbers of outcome events or summary measures<br><br>**Tables 1-2.**
Main results	16	(*a*) Give unadjusted estimates and, if applicable, confounder-adjusted estimates and their precision (eg, 95% confidence interval). Make clear which confounders were adjusted for and why they were included

Table 3-4.

Other analyses	17	Report other analyses done—eg analyses of subgroups and interactions, and sensitivity analyses<br><br>**Pages 13 and 14 of manuscript (Results). Supplementary Table S1-S8**
**Discussion**		
Key results	18	Summarise key results with reference to study objectives<br><br>**Pages 15-18 (Discussion)**
Limitations	19	Discuss limitations of the study, taking into account sources of potential bias or imprecision. Discuss both direction and magnitude of any potential bias<br><br>**Pages 15-18 (Discussion)**
Interpretation	20	Give a cautious overall interpretation of results considering objectives, limitations, multiplicity of analyses, results from similar studies, and other relevant evidence<br><br>**Pages 15-18 (Discussion)**
Generalisability	21	Discuss the generalisability (external validity) of the study results<br><br>**Pages 15-18 (Discussion)**

*Give information separately for cases and controls in case-control studies and, if applicable, for exposed and unexposed groups in cohort and cross-sectional studies.

Note: An Explanation and Elaboration article discusses each checklist item and gives methodological background and published examples of transparent reporting. The STROBE checklist is best used in conjunction with this article (freely available on the Web sites of PLoS Medicine at http://www.plosmedicine.org/, Annals of Internal Medicine at http://www.annals.org/, and Epidemiology at http://www.epidem.com/). Information on the STROBE Initiative is available at www.strobe-statement.org.
